# Involvement of the *BDNF* Gene in Loneliness in Adolescence: A Report of Opposite Gene Effects in Boys and Girls

**DOI:** 10.1371/journal.pone.0092768

**Published:** 2014-03-19

**Authors:** Maaike Verhagen, Eeske van Roekel, Rutger C. M. E. Engels

**Affiliations:** 1 Behavioural Science Institute, Radboud University Nijmegen, Nijmegen, The Netherlands; 2 Interdisciplinary Center of Psychopathology and Emotion Regulation, University Medical Center Groningen, University of Groningen, Groningen, The Netherlands; Radboud University, Netherlands

## Abstract

Previous research has shown that loneliness has a heritable component and that genes within the serotonin-, dopamine-, and oxytocin systems are related to loneliness in adolescence. In the present study, the relation between the *BDNF* Val66Met polymorphism and loneliness in adolescent boys and girls was examined in a longitudinal study spanning five annual waves (*N* = 305). Latent growth curve modeling (LGCM) was used to examine the baseline level and the change in loneliness over time. The main finding was that the *BDNF* gene was not related to loneliness in the total sample. A *BDNF* by sex interaction was found, in that Met carrying girls had the highest levels of loneliness at baseline, whereas in boys the ValVal genotype was related to higher levels of loneliness. Our results underline the importance of sex-stratified analyses when examining effects of the *BDNF* genotype and the necessity of conducting gene studies to intermediate phenotypes of loneliness.

## Introduction

Humans are born with a fundamental need to bond with others, which is hypothesized to be the first need after certain needs such as food and safety are fulfilled [1]. Loneliness is a condition that arises when this need to belong is not fulfilled, and is defined as the negative emotional response to a perceived discrepancy between the actual and desired quantity and quality of one’s social network [2]. Several studies have shown that loneliness impacts on cognitions in various ways [3]–[5]. It has been established that feeling socially isolated leads to a hypervigilance for environmental threats, hereby affecting attention and memory processes [4]. These cognitive biases influence (social) behaviors in some sort of self-fulfilling way, in that eventually thoughts and cognitions are being confirmed by behavioral patterns. A vicious circle of cognitions and behaviors might be construed that attenuates or maintains individual levels of loneliness (see also figure 3 in [3]). Simply stated, the negative ideas and expectations that lonely people hold about others, together with their better memory for negative events may lead to negative behavioral interaction patterns through which negative schemes about themselves are being confirmed. Cacioppo et al. [3] stated that the forthcoming alterations in the nature of social interactions and the enhanced threat awareness in social contexts might activate neurobiological mechanisms that increase HPA axis functioning. It has further been suggested that brain-derived neurotrophic factors (BDNF) might play a role in this relationship between cognitive processes and loneliness, however, the exact mechanism is unclear. The possibility of involvement of BDNF in loneliness is further illustrated by an animal study showing substantial down regulation of BDNF in rat brains after a social isolation procedure [6]. As the expression of BDNF is regulated by the brain derived neurotrophic factor (*BDNF*) gene, we aimed to examine whether the *BDNF* gene is related to loneliness in adolescence.

The *BDNF* gene is located on chromosome 11p13. A single nucleotide polymorphism (SNP) in exon 11 of the *BDNF* gene (rs6265) results in an amino acid substitution from valine to methionine at codon 66 (Val66Met) in the prodomain of the gene [7]. As stated above, the *BDNF* gene regulates the expression of BDNF which play an important role in neuronal plasticity and connectivity in the adult brain [8], [9]. BDNF are important in processes such as the proliferation, differentiation, and survival of neuronal cells [7], [9], [10]. As the hippocampus is the brain site in which the expression of neurotrophic factors is highest [11], several *BDNF* candidate gene studies in relation to structural and functional aspects of the hippocampus have been conducted.

Structural MRI studies showed associations of the Val66Met variant with hippocampal volume [12]–[15], with Met allele carriers having smaller bilateral hippocampal volumes. Next to this, structural amygdala alterations have also been reported (smaller volumes in Met carriers [16]–[18]). In addition to structural brain alterations, functional MRI (fMRI) studies in both adults and adolescents have shown involvement of the Val66Met variant in amygdala and hippocampal brain activity during memory and emotion processes [18]–[25]. These studies are important since they provide insight in the relation between the Val66Met variant and cognitive and emotional processes which could be considered intermediate measures for loneliness. It was shown that Met allele carriers showed decreased engagement or hippocampal activity during memory tasks, compared to ValVal homozygotes [20]. From another fMRI study, using emotional valenced words, it appeared that the Met allele was associated with increased encoding activity for negative words in the hippocampus [21]. Experiments in which emotional and neutral pictures were displayed showed that Met allele carriers had stronger activation in the (right)amygdala when viewing emotional stimuli compared to neutral pictures (in females) [22], [24]. During fear processing, Met carriers showed stronger activation in multiple brain parts, including the anterior cingulate cortex, and a decreased functional connectivity with the hippocampus [23]. The only fMRI study conducted in adolescents that examined the association of this *BDNF* SNP in relation to emotionally laden stimuli is important to mention here [26]. A significant association of the Met allele with neural activity in the amygdala and hippocampus (when viewing emotional faces) was observed in adolescents with depression or anxiety, but not in healthy controls. These results suggest involvement of the *BDNF* gene in affective emotion processing as well as in memory processes (e.g. encoding and retrieval), measures that might well underlie loneliness. In sum, one could suggest that Met carriers display a higher sensitivity for negative or emotional stimuli. Because lonely people encode social experiences more thoroughly, recall social experiences more easily [4] and show a hypervigilance for threats or negative stimuli, this gene needs to be explored in relation to loneliness.

An issue that should be mentioned here is that some studies did not find *BDNF* associations for the total group, but did reveal sex-specific findings. For example, a meta-analysis on depression [27] showed a significant Val66Met effect in males; only male Met carriers had an increased risk of depression. In line with this, an fMRI study showed sex-specific associations for the amygdala; the Met allele was associated with memory formation in males only [28]. Further, a stress response task showed that female Met carriers displayed the strongest cortisol response during a stress task [29]. This might indicate that the *BDNF* gene has sex-specific effects contributing to brain dimorphisms or the development of disorders and could also influence loneliness in a sex-specific manner.

The aim of the current study was to examine (sex-specific) relations between *BDNF* genotype and the onset and change over time in loneliness in adolescence. This is the first study examining the *BDNF* gene in relation to loneliness. Given the Val66Met results from imaging studies in relation to affective emotion processing and memory processes, which are involved in the susceptibility for loneliness, we hypothesized that adolescents with one or two Met alleles would have higher levels of loneliness than adolescents with the ValVal genotype. Given the sex differences in the effects of *BDNF* genotypes [27]–[29], we also examined *BDNF* by sex interactions. We did not have a specific hypothesis for this interaction as sex-findings are not unequivocal.

## Methods

### Procedure

The present study used data from the *Family and Health* study, which aimed to examine different socialization processes in relation to health behaviours [30] in Dutch adolescents and their parents. Data were collected in five annual waves. Approval was obtained from the Central Committee on Research Involving Human Subjects on collecting the data. Written informed consent was obtained from parents and adolescents. For a more detailed description of the Procedure, see van Roekel and colleagues [31].

### Participants

The sample in the present study consisted of 305 adolescents, of which 53.8% were girls. Ages at the first wave ranged between 13 and 15, with a mean age of 13.35 (*SD* = .51). Educational levels were equally distributed, with one third of the adolescents attending lower-level education (preparatory secondary school for technical and vocational training), one third attending middle-level education (preparatory secondary school for college) and one third attending higher-level education (preparatory secondary school for university). We conducted attrition analyses to examine whether adolescents who gave their consent for genotyping (*n* = 305) differed from adolescents who did not (*n* = 123). Results from the *T*-tests showed no significant difference between dropouts and participating adolescents in age and levels of loneliness. Chi square statistics revealed that participating adolescents and dropouts did not differ in sex (*χ^2^*[428] = .89, *p* = .20) or educational level (*χ^2^*[422] = 9.84, *p* = .08).

### Measures

#### Loneliness

Loneliness was measured at all time points with a subscale from the Louvain Loneliness Scale for Children and Adolescents (LLCA) [32]. This scale consisted of 12 items, measured on a 4-point scale (1 = never; 4 = always). A sample item is ‘I feel abandoned by my friends’. Participants’ scores ranged from 12 to 48, with higher scores indicating higher levels of loneliness. Cronbach’s alpha varied from.91 to.94 over the five measures.

#### BDNF genotype

DNA was isolated from saliva using the Oragene system (DNA Genotek Inc., Kanata, Ontario, Canada). The *BDNF* rs6265 polymorphism was genotyped using Taqman analysis. For this polymorphism a readymade Taqman Allelic Discrimination assay was ordered (ID: *BDNF* (rs6265), C__11592758_10, reporter 1: VIC-C-allele, reverse assay, Applied Biosystems, Nieuwerkerk a/d IJssel, The Netherlands). The genotyping was carried out in a volume of 5 μl containing 10 ng of genomic DNA, 2.5 μl of Taqman Mastermix (2x; Applied Biosytems) and 0.0625 μl of the Taqman assay (40x) and 1.4375 μl of MilliQ. The amplification for the Taqman Allelic Discrimination assay (C__11592758_10) was performed by an initial denaturation at 95°C for 12 min, followed by 40 cycles of denaturation at 92°C for 15 seconds and annealing/extension at 60°C for 1 minute, this was carried out on a 7900 Fast Real-Time PCR System. Genotypes were scored using the algorithm and software supplied by the manufacturer (Applied Biosystems). Generally, 5% blanks as well as duplicates between plates were taken along as quality controls during genotyping. Genotyping was performed in a CCKL-accredited laboratory at the Department of Human Genetics of the Radboud University Nijmegen Medical Centre in Nijmegen. No deviations from Hardy-Weinberg equilibrium (HWE) were detected (p = .96). We dummy-coded the *BDNF* genotype into 0 (ValVal) and 1 (ValMet and MetMet) to maximize the power of the analyses [13].

### Statistical Analyses

We used latent growth curve modeling (LGCM) to estimate both the baseline level of loneliness (i.e. intercept) and the rate of change over time (i.e. slope) [33]. LGCM is an excellent way to examine individual variation in the development of loneliness and to investigate whether certain predictors relate to these changes over time. All analyses were conducted in Mplus, a statistical software program specifically designed for structural equation modeling analyses [34]. Parameters in the models were estimated by applying the maximum likelihood estimator with robust standard errors (MLR), which is required when dependent variables have non-normal distributions. In the first step, the empty model without predictors was tested to estimate the baseline level of loneliness and the rate of change over time. Second, the *BDNF* genotype was entered as a predictor in the model, while controlling for sex. Third, we examined whether the interaction between sex and the *BDNF* genotype was related to the intercept or slope of loneliness. We calculated the proportion of explained variance (ΔR^2^) by subtracting the r square value of the previous model (without the predictor) from the r square value of the present model (with the predictor).

## Results

### Descriptive Statistics

**Figure 1:**
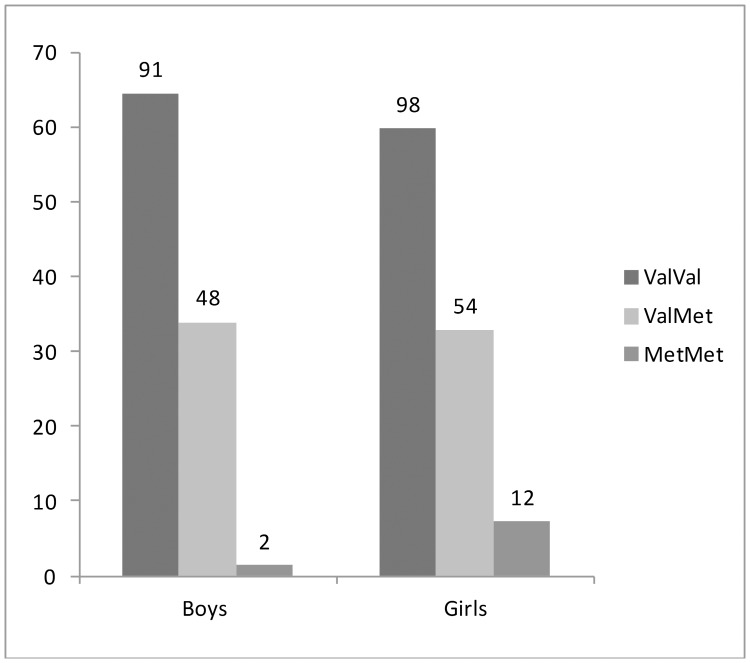
Genotype distribution split for boys and girls.

The genotype frequencies for the *BNDF* genotype split by sex can be found in Figure 1. Means, standard deviations, and correlations between model variables are depicted in Table 1, separately for boys and girls. Mean levels of loneliness are relatively low considering the range (12–48), but are comparable to other community samples [35]. For girls, a small correlation was found between *BDNF* genotype and loneliness at T2, indicating that Met carriers had higher levels of loneliness at T2. For boys, no significant correlations were found between *BDNF* genotype and the loneliness measures. The five loneliness measurements were moderately to highly correlated (from *r* = .37 to *r* = .73).

**Table 1:** Correlations Among Model Variables Split for Boys and Girls.

Variable	*M (SD)* Boys	*M (SD)* Girls	1	2	3	4	5	6
1. *BDNF* a	.35 (.48)	.40 (.49)	–	.11	.16*	.01	.07	.04
2. Loneliness (T1)	19.31 (6.41)	18.35 (6.75)	−.12	–	.53***	.50***	.38***	.53***
3. Loneliness (T2)	18.15 (6.45)	19.09 (6.46)	−.13	.63***	–	.56***	.47***	.39***
4. Loneliness (T3)	18.44 (6.71)	18.06 (6.20)	−.11	.57***	.68***	–	.52***	.56***
5. Loneliness (T4)	17.84 (6.33)	18.43 (6.43)	−.11	.45***	.47***	.55***	–	.67***
6. Loneliness (T5)	17.01 (6.42)	18.26 (6.85)	−.11	.44***	.38***	.37***	.73**	–

*Note. BDNF* = Brain Derived Neurotrophic Factor gene. Correlations for girls are depicted above the diagonal, below the diagonal the correlations for boys.

a 0 = ValVal; 1 = ValMet and MetMet.

*p<.05. **p<.01. ***p<.001.

### Model Findings

**Figure 2:**
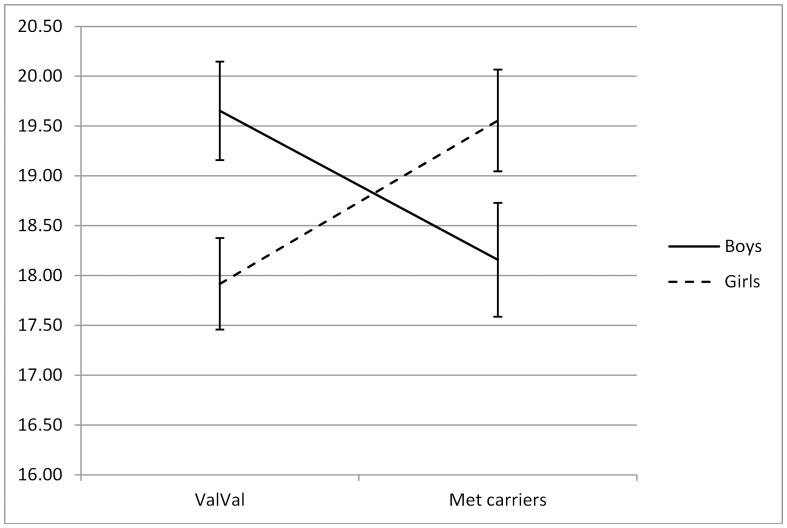
Interactions between *BDNF* genotype and sex on intercept of loneliness.

First, the model without predictors was tested (Table 2). Both the intercept and slope were significant, indicating that adolescents scored on average 18.83 on loneliness at baseline and significantly decreased in loneliness over time. Second, sex was entered in the model. Results showed a significant relation between sex and the slope of loneliness, in that girls remained relatively more stable in loneliness over time than boys. This effect explained 3% of the variance of the slope. No effect was found on the intercept. In the third model, the relation between *BDNF* genotype and loneliness was examined. No relations were found between the genotype and intercept or slope. Fourth, the interaction between *BDNF* genotype and sex was examined in relation to loneliness. A significant relation was found with the intercept of loneliness (see Figure 2). Boys carrying the ValVal genotype had higher levels of loneliness at baseline than boys carrying a Met allele, whereas in girls, Met allele carriers had the highest levels of loneliness at baseline. The interaction between the *BDNF* genotype and sex explained 2.5% of the intercept variance. No effects were found on the slope of loneliness.

**Table 2:** Regression of Initial Level (Intercept) and Rate of Change (Slope) in Adolescents’ Loneliness on BDNF Genotype.

Predictor	Intercept	ΔR^2^	Slope	ΔR^2^	?^2^ (*df*)	CFI	RMSEA
**1.** Initial developmental model	18.83**		−.28**		40.30 (10)	.91	.10
**2.** Sex	−.05 (.07)	.00	.18 (.08)*	.03	48.53 (13)	.91	.095
**3.** *BDNF*	.02 (.07)	.00	−.09 (.09)	.01	52.63 (16)	.91	.087
**4.** *BDNF* × Sex	.68 (.27)*	.03	−.17 (.35)	.00	56.67 (19)	.92	.081

*Note. BDNF* = Brain Derived Neurotrophic Factor gene.

*p<.05. **p<.01. ***p<.001.

In all analyses, we controlled for sex. Only new variables entered in the model are depicted in the table.

## Discussion

The goal of the present study was to examine relations between *BDNF* Val66Met genotype and loneliness in adolescence. As no previous studies have described the link between *BDNF* and loneliness, our hypothesis was based on literature on proxy measures for loneliness, e.g. affective and cognitive information processes. These have been associated with loneliness and the *BDNF* gene before. The *BDNF* Val66Met variant is a reasonable candidate gene since the hippocampus, which plays a role in these cognitive processes, is the most important site of action for BDNF. We hypothesized that adolescents with one or two Met alleles would have the highest levels of loneliness. Our main finding was that the *BDNF* gene was not related to loneliness in the total sample and that opposite gene effects were observed for girls and boys in baseline levels of loneliness.

For girls, the results were in line with our hypothesis and with fMRI studies in females showing that the *BDNF* Met allele is associated with more activation in the amygdala when viewing emotional pictures compared to ValVal homozygote females [22], [24]. This was suggested to be an indication for Met allele carriers being more reactive to emotional stimuli [22], [24]. This has also been shown in a study in which hippocampal activation was measured in a task comparing negative words to neutral words. Met carriers showed stronger activation during the negatively valenced words compared to neutral words [21]. This is in accordance with the finding that lonely people display higher attention to vocal emotional tones than nonlonely people [4]. Thus, the results from fMRI studies on attention and emotion processes are in line with theory stating that lonely people might have higher sensitivity for social and emotional cues [36], [37] in order to restore belongingness levels [4]. Our finding with loneliness might illustrate that this is especially apparent in girls carrying a Met-allele. Despite the effect sizes being relatively small, it should be noted however, that these are reasonably common for candidate gene studies [38].

It has also been suggested that the *BDNF* Met allele is associated with a diminished capacity to process emotional environmental cues and diminished learning from previous emotional experiences and hereby, a lowered ability to respond appropriately to those cues [23], [24]. This could place subjects at higher risk for experiencing loneliness as this decreased ability prevents them from social inclusion. This could explain why girls carrying a Met allele experience higher levels of loneliness.

Despite several genetic associations with the Met allele, associations with the Val allele have also been described. In this study, we found slightly higher loneliness scores at baseline in boys with the ValVal variant. Previous candidate gene studies suggested associations between the ValVal genotype and higher levels of trait anxiety [39] and neuroticism [40], both phenotypes being closely related to loneliness [41]. Further, although most imaging studies found effects for stronger brain arousal in Met carriers, one study described more activation in ValVal subjects in response to faces displaying negative emotions [19]. In general, most sex-specific *BDNF* gene analyses described associations of the Met allele in males only [27], [28]. However, these studies were conducted in adult samples. The only *BDNF* gene study examining sex differences in adolescents did not find significant effects [26]. However, this might be due to their limited power to find significant effects. As estrogen receptors interact with BDNF [42], it might well be that in adolescent samples, sex differences are at play.

The contrasting results in girls and boys in our study are similar to a study on cortisol responses that found that male college students with the ValVal genotype showed a higher cortisol response in reaction to the Trier Social Stress Test than ValMet carriers. An opposite effect was observed in females [29]. As higher levels of cortisol are related to momentary feelings of loneliness [43], [44], this possibly explains why similar sex differences were found in the study on cortisol responses [29] and in the present study on loneliness.

An important study to genetic sex-specificity of quantitative traits suggested that even in the absence of differences on trait levels between the sexes, genes may act differently in both sexes, either through sex-linked genes or through interaction with gonadal hormones [45]. The sex-specific gene effects observed in our study may be due to hormonal differences that influence gene expression. For example, it is known that estrogen can influence the level of this neurotrophic factor (BDNF) in the brain [42], [46], [47]. This is illustrated by a mice study showing sex differences in depressive behaviour in *BDNF* knock-out mice, in that male knock-out mice show normal levels of depression-like behaviour, whereas female knock-out mice display higher levels of depression-like behaviour [48]. An addition to this is the finding that despite similar task performance during functional imaging sessions in males and females, different underlying gene effects [28] or differential activation patterns in neural substrates [49] were identified. Thus, gene effects and brain activation differed between males and females in the absence of observable differences on a behavioral level. This stresses the importance of examining sex differences to detect subtle underlying individual variation. So far, studies have shown sex-specific *BDNF* gene effects, but future studies should try to disentangle the underlying mechanism.

An additional point of attention is that depression research showed that BDNF effects are diverse in the brain, might be regionally specific [8], [10], [50], [51], and subsequently, that the *BDNF* gene has been associated with various phenotypes that are interrelated with loneliness such as trait anxiety [39] and depression [27]. This is a common phenomenon, also known as multifinality [52]. This indicates that a genetic marker can be associated with related, though different, behavioral outcomes depending on individual patterns of adaptation. Therefore it is important in *BDNF* gene studies to consider which brain parts might be involved in the etiology of the disease process or trait of interest. A first step to accomplish this could be by conducting genetic imaging studies in which the *BDNF* gene is examined in relation to specific brain endophenotypes for certain traits [53]. It could well be that internalizing disorders share underlying endophenotypes that account for the association with the *BDNF* variant. With regards to internalizing disorders, one could think of intermediate endophenotypes such as biased information processing of social cues. In general, for a better understanding of sex-specific gene effects in disorders and traits, it is essential to relate the gene to brain function or structure [53]. Thus, based on brain structure and function and biological processes involved in the etiology of the trait of interest, one could examine how the *BDNF* gene contributes to the phenotype of interest.

To place our findings in a broader context, it is also important to realize that loneliness is polygenic in nature, implying that multiple genes influence this phenotypic outcome. Next to the *BDNF* gene, previous studies have examined the oxytocin receptor gene (*OXTR*) [54]–[56], the dopamine receptor gene (*DRD2*) [57], the serotonin receptor gene (*5-HTTLPR*) [31] and the corticotrophin-releasing hormone receptor 1 gene (*CRHR1*) [58] in relation to several loneliness measures in adolescents, adults and elderly. Both genetic main effects were observed [31], [54]–[56] and gene by environment interactions (the environment mostly defined as support from others) [31], [56]–[58]. It would be very exciting to have a more detailed insight in the interplay of these different biological systems. Up to date, it is largely unknown if and how different systems exactly interact. Shortly stated, what we do know is that both the *BDNF* and *OXTR* genes are involved in modulating or regulating the stress response [59], [60]. However, the fact that both genes might be implicated in stress response systems is no evidence for, and does not shed light on how the biological brain systems that underlie the genetic markers, are in any way related on a molecular level and influence levels of loneliness. Future candidate gene and gene by environment studies could shed light on the interplay of different genes involved in loneliness.

The present study has some limitations that should be mentioned. First, our sample was a normative group, consisting of healthy adolescents living in intact families. This resulted in relatively low loneliness scores. Future studies could consider examining the relations in other groups, such as in clinical samples, in which loneliness scores are higher. Second, the present study was the first to examine the *BDNF* genotype in relation to loneliness, hence replication of our results is important. A limitation related to this is that, in so far, no literature has described the influence of this gene on loneliness. Therefore, we based our hypotheses and explanations on related measures (e.g. affective information processing), which we presume to be possible intermediate phenotypes. Another limitation is that our sample size was relatively small. In genetic studies, sample sizes with sufficient statistical power are required to detect true genetic associations. However, we cautiously consider our sample to be adequately powered to detect genetic associations. Power calculations conducted with Quanto [61] based on 80% statistical power, an alpha of.05 and 3% explained variance, revealed that a sample size of 247 adolescents was required. Next to this, the longitudinal design with measures at multiple time points increased the power of our analyses [62], [63]. As this study was conducted in adolescents, the results cannot be generalized to other age groups. Even more, age-effects may play a role: Developmental changes in the expression of the *BDNF* gene have been described [64]. This is illustrated by a study to rumination and depression in mothers and their daughters which revealed an inverse association: ValVal was associated with depression in children, whereas in mothers depression was associated with the ValMet genotype [65]. This might indicate that biological developments influence BDNF effects on behavioral measures.

The main finding of the present study was that the *BDNF* genotype had no effect in the total group of adolescents, but did have different effects on baseline levels of loneliness in boys and girls. These results underline the importance of sex-stratified analyses when examining effects of the *BDNF* genotype, even in the absence of clear behavioral differences.
